# One Tool for Many Jobs: Divergent and Conserved Actions of Androgen Signaling in Male Internal Reproductive Tract and External Genitalia

**DOI:** 10.3389/fendo.2022.910964

**Published:** 2022-06-30

**Authors:** Ciro M. Amato, Humphrey H-C. Yao, Fei Zhao

**Affiliations:** ^1^ Reproductive Developmental Biology Group, National Institute of Environmental Health Sciences, Research Triangle Park, NC, United States; ^2^ Department of Comparative Biosciences, School of Veterinary Medicine, University of Wisconsin-Madison, Madison, WI, United States

**Keywords:** androgen, androgen receptor (AR), external genitalia (ExG), wolffian duct, penis, masculinization

## Abstract

In the 1940s, Alfred Jost demonstrated the necessity of testicular secretions, particularly androgens, for male internal and external genitalia differentiation. Since then, our knowledge of androgen impacts on differentiation of the male internal (Wolffian duct) and external genitalia (penis) has been drastically expanded upon. Between these two morphologically and functionally distinct organs, divergent signals facilitate the establishment of tissue-specific identities. Conversely, conserved actions of androgen signaling are present in both tissues and are largely responsible for the growth and expansion of the organs. In this review we synthesize the existing knowledge of the cell type-specific, organ specific, and conserved signaling mechanisms of androgens. Mechanistic studies on androgen signaling in the Wolffian duct and male external genitalia have largely been conducted in mouse model organisms. Therefore, the majority of the review is focused on mouse model studies.

## Introduction

Androgen signaling pathway is essential for development of male organs across vertebrate animals (1). This pathway is responsible for the growth and differentiation of the prostate, penis, epididymis, vas deferens and seminal vesicle during embryonic development for normal structural development and function. Since the 1940s, we have known that the loss of testis-derived androgens disrupts male reproductive development. XY embryos without the testes develop into female sexual characteristics due to the absence of male-specific reproductive structures (2, 3).

Due to the dependency on androgen signaling, male reproductive structures are particularly sensitive to disrupted fetal development and adulthood dysfunction (4, 5). The incidence rate of children born as intersex, a condition where patients contain a combination of male and female reproductive organs, is relatively common, with incidence ranging from 0.37-1.7% cases per year (6). The intersex spectrum includes defects of the penis (e.g. hypospadias and micropenis) and defects in the reproductive tract (e.g. cryptorchidism). Often times the androgen-related congenital defects of the external genitalia and Wolffian duct lead to compromised reproductive functions and infertility.

In this review, we discuss the diverse and conserved androgen actions in two critical reproductive organs, the Wolffian duct-derived tissues and the male external genitalia, specifically the penis, during embryonic development. We review the literature with the goal of understanding the pleiotropic nature of AR. To do this, we tease apart the cell populations of each organ. Then we investigate the androgen signaling programs that are specific to each cell population. By reviewing the literature in this way, we can begin to discern the molecular determinants that specify the distinct androgen signaling programs in each cell population. To further understand the general functions of AR, we discuss common androgen signaling events in both the Wolffian duct and male external genitalia. Because our knowledge about androgen signaling in male sexual differentiation derive largely from mouse studies, the majority of the following sections focus on mouse models. Reviewing both the internal and external reproductive tract provides a roadmap for future studies and allows us to investigate the many diverse and conserved action of androgen signaling.

## Mechanisms of Androgen Signaling Pathway

Androgen signaling pathway is mediated by the androgen receptor (AR), which mainly acts as a ligand-activated transcriptional factor (7). Loss of function mutations of AR in mice lead to feminization of internal reproductive tract organs and external genitalia even though the fetal production of androgens is unaffected (8). Human XY individuals with androgen insensitivity syndrome caused by disruptions in the androgen signaling cascade develop phenotypically female internal and external genitalia (9). Conversely, XX patients with congenital adrenal hyperplasia, a disorder characterized by excessive production of androgens in adrenal glands, develop masculinized external genitalia (10). Thus, androgens are the driving force for male sexual differentiation.

Androgens are first produced by the Leydig cells in the fetal testis, in which express the key steroidogenic enzymes for androgen production Cytochrome P450 17a1 (*Cyp17a1*) and 3β-Hydroxysteroid (3β-HSD) (11). These two enzymes and other enzymes in the cascade of steroidogenesis convert cholesterol to androgens (11, 12). The testis-derived androgens then enter the blood circulation and reach the target organs. Once androgen reaches an organ that expresses AR, it passively diffuses through the cell membrane and either bind directly to AR in the cytosol, or be converted into a more potent androgen, dihydrotestosterone, by the 5-α reductase (SRD5a1 and SRD5a2) enzymes (13). Binding of testosterone or dihydrotestosterone to AR triggers the release of AR from molecular chaperones and translocation of AR to the nuclei, where it regulates target gene expression.

In addition to the classic genomic action, binding of androgens to AR in the cytoplasm initiate signal transduction pathways to modulate cellular proliferation and migration, which is also known as non-genomic actions of the AR (14). When the DNA-binding domain, which transmit the genomic action of AR, is removed from AR, male mice display a complete androgen insensitive phenotype with feminized external genitalia and loss of internal reproductive tracts (Wolffian duct and prostate) (15). These results underscore the genomic actions of the AR are indispensable for normal male sexual differentiation. Another study generated transgenic mice expressing mutant *Ar* with only genomic or non-genomic actions in the absence of endogenous AR (16). They found that seminal vesicle and epididymis are degenerated in both mutant mice, suggesting both genomic and non-genomic actions are required for normal development of Wolffian duct-derived tissues. On the other hand, main actions of AR in external genitalia are mediated through the genomic actions based on the observation that mice with a mutated non-genomic region of *Ar* develop normal penises (17).

## Heterogeneity of Androgen Actions in the Wolffian Duct

### Ontology of Wolffian Duct-Derived Organs

Wolffian ducts are paired embryonic structures that give rise to male internal reproductive tract organs including the epididymis, vas deference and seminal vesicles (18). In both XY and XX mice embryos, the Wolffian duct derives from the intermediate mesoderm at around embryonic day 8.5 (E8.5) and elongates craniocaudally till it reaches the cloaca by E9.5 (19). The Wolffian duct is surrounded by its mesenchyme, which expresses AR first on E12.5 in both sexes (20, 21). In XX embryos, where ovaries do not produce androgens, AR action does not take place, leading to degeneration of Wolffian ducts (18). In addition to the lack of AR action, Wolffian duct degeneration in XX embryos requires COUP-TFII (Chicken ovalbumin upstream promoter transcription factor II or NR2F2), an orphan nuclear receptor specifically expressed in the Wolffian duct mesenchyme (22). On the other hand, in XY embryos, fetal testes produce androgens starting from E12.5 (23). Under the influence of testis-derived androgens, the Wolffian ducts are stabilized and then undergo regionalization and differentiation into the epididymis, vas deferens and seminal vesicle in a craniocaudal fashion.

### Mesenchymal AR Actions Are Critical for Wolffian Ducts Differentiation

Although the AR is expressed in both the epithelium and mesenchyme during Wolffian duct differentiation, AR action in the mesenchyme appears to govern fetal Wolffian duct development. Ablation of AR in the Wolffian duct epithelium does not affect Wolffian duct maintenance or morphogenesis (20). The specific role of AR in Wolffian duct mesenchyme has not been determined directly by mesenchyme-specific AR knockout model; however, classic tissue recombination studies imply that the mesenchymal AR actions govern androgen-induced epithelial morphogenesis. For example, the epithelium of upper Wolffian ducts (future epididymis) develops seminal vesicle-like structures when combined with lower Wolffian duct mesenchyme (prospective seminal vesicle) (24). The urogenital sinus is another androgen target tissue, which undergoes prostatic morphogenesis upon androgen actions but develops vagina-like morphology in the absence of androgen. When wild-type mesenchyme is grown adjacent to either wild-type or Tfm (testicular feminized mice that lacks functional AR) epithelium, the urogenital sinus still undergoes prostatic morphogenesis. In contrast, when urogenital sinus epithelium is recombined with the mesenchyme from Tfm embryos, the epithelium develops vagina-like structures (25). These results demonstrate that mesenchymal AR actions dictate Wolffian duct differentiation.

### How Do Mesenchymal AR Actions Induce the Stabilization of Wolffian Ducts?

One possible signal downstream of mesenchymal AR actions in promoting Wolffian duct survival is epidermal growth factor (EGF), which regulates a wide range of cellular events including cell survival and proliferation (26). *Egf* expression is elevated in the whole mesonephric tissue during fetal male reproductive tract differentiation and flutamide (AR antagonist) exposure during sexual differentiation decreased *Egf* in male mice embryos (27). Receptors for EGF or EGFR is an integral membrane tyrosine kinase that is activated upon binding of multiple ligands including EGF. Expression of EGFR is also induced by testosterone and inhibition of EGFR using an anti-EGFR antibody blocks Wolffian duct growth in cultured murine mesonephros (28). Global *Egfr* knockout in mice causes genetic background dependent placental abnormalities and embryonic lethality before E11.5, preventing analysis of Wolffian duct development in male embryos (29). *Egf* knockout mice have normal phenotype (30), suggesting other ligands for EGFR might compensate for the loss of *Egf* in Wolffian ducts.

To promote Wolffian duct stabilization, mesenchymal AR actions need to antagonize inhibitory effects of another mesenchymal transcriptional factor, COUP-TFII. COUP-TFII is an orphan nuclear receptor expressed in the mesenchymal compartment of many developing organs (31, 32), including the mesonephros in XX and XY embryos (33). In XX embryos, the absence of *Coup-tfII* increased expression of two mesenchymal growth factors *Fgf7* and *Fgf10*, which activated their receptor FGFR2 in Wolffian duct epithelium to promote Wolffian duct survival in XX embryos in the absence of AR actions (22). These results suggest that under normal conditions, COUP-TFII action in the Wolffian duct mesenchyme is to inhibit Wolffian duct survival by suppressing FGF signaling. In XY embryos, to promote the stabilization of Wolffian ducts, the mesenchymal AR is speculated to induce the EGFR-mediated signaling pathway in Wolffian duct stabilization, therefore, providing another survival signal different from *Fgfs* that are suppressed by COUP-TFII. Genome-wide binding of COUP-TFII in the XX and XY mesonephros reveals that COUP-TFII binding motif is different from established AR binding motifs, providing another evidence that these two mesenchymal transcriptional factors target differential genes in regulating Wolffian duct survival (34).

### How Do Mesenchymal AR Actions Induce Region-Specific Gene Expression and Differentiation in Wolffian Ducts at the Fetal Development?

**Figure 1:**
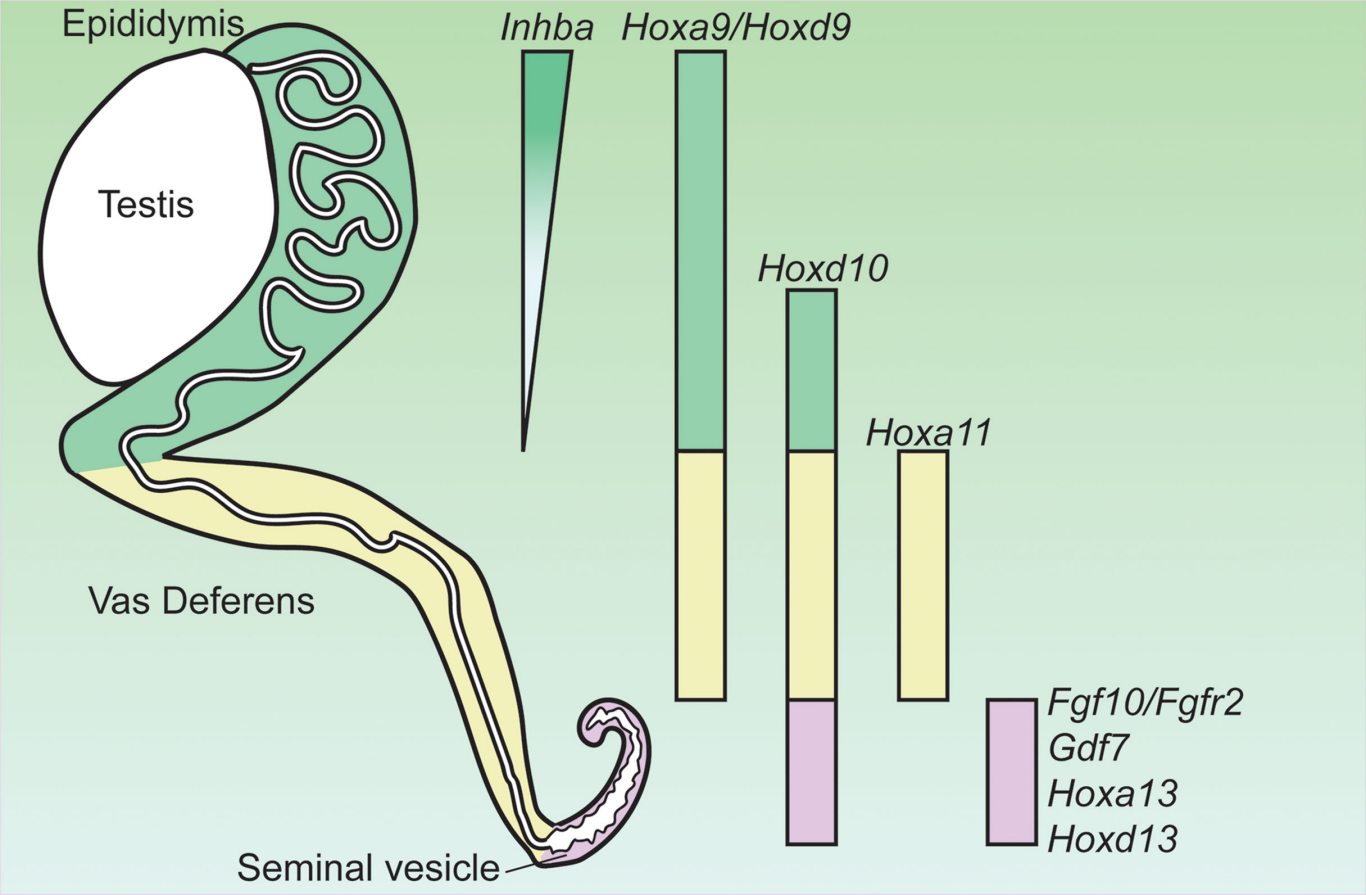
Expression of genes critical for the regionalization of the Wolffian duct and potentially involved in region-specific androgen actions. Hox, homeobox; Inhba, inhibin β A; Gdf7, growth differentiation factor 7; Fgf10, fibroblast growth factor 10; Fgfr2, fibroblast growth factor receptor 2. Adapted from (35).

AR actions in the mesenchyme drive the differentiation of the Wolffian duct into three morphologically and functionally distinct organs: the epididymis, vas deferens and seminal vesicle during fetal development from rostral (i.e. anterior) to caudal (i.e. posterior) regions (**Figure 1**). Regionalization of the Wolffian duct along the rostral-caudal axis is regulated by region-specific expressed HOX genes in the epididymis (*Hoxa9* (36), *Hoxd9* (36) and *Hoxa10* (37), vas deference (*Hoxa10* (37) and *Hoxa11* (38)), and the seminal vesicle (*Hoxa13* and *Hoxd13*) (39) (**Figure 1**). Deletion of these region-specific Hox genes can often cause homeotic transformations. For example, *Hoxa10* mutant males have anterior transformation of the cauda epididymis and the proximal vas deferens (37, 40). The vas deferens in either individual *Hoxa11* knockout or *Hoxa11* and *Hoxd11* (a paralog to *Hoxa11*) double males (38, 41) resemble an epididymis. Seminal vesicles become hypoplastic in both *Hoxd13^-/-^* and *Hoxa13* ^+/–^; *Hoxd13* ^–/–^ compound mutant mice (42). In addition to *Hox9-13*, additional region-specific *Hox* transcripts, such as *Hoxc4*, *Hoxc6*, *Hoxc9* have been identified in the epididymis by comparing gene transcriptional profiles of the epididymis, vas deferens and efferent duct at E14.5, E16.5, E18.5 and P1 (43). Although roles of *Hox* genes in the regionalization of the Wolffian duct is well recognized, it is still unknown how these *Hox* transcription factors interact with AR actions and determine region-specific AR actions.

Mesenchymal AR actions induce region-specific paracrine mesenchymal growth factors for regulating epithelial morphogenesis in Wolffian duct differentiation. In the rostral (anterior) region, AR action promotes epididymal coiling which relies on the action of inhibin β A (*Inhba*) (44). *Inhba* is a critical component of inhibins and activins (members of TGFβ superfamily ligand) and is highly expressed in the mesenchyme of the anterior Wolffian duct before the initiation of epididymal morphogenesis (**Figure 1**) (44). Androgen partially increased *Inhba* expression after E13.5 based on *ex vivo* organ culture studies (44). Male embryos lacking *Inhba* fail to develop epididymal coiling due to a dramatic decrease in epithelial proliferation even if AR expression is intact (44).

In addition to INHBA, mesenchyme-derived WNT ligands or WNT signaling activators may also play a role in epididymal morphogenesis. The WNT signaling pathway is an evolutionarily conserved pathway that regulates organogenesis (45). The WNT signaling pathway includes the extracellular WNT ligands, which stimulate intracellular signal transduction cascades to regulate gene expression and cellular differentiation in target cells (46). Two intracellular pathways in the target cells mediate WNT signaling: β-catenin (CTNNB1)-dependent or -independent pathways (46). The WNT/β-catenin signal also depends on the Wnt signaling activators, R-spondins (RSPOs), which elicit their functions by binding to the WNT co-receptors (*Lgr4-6*, the leucine-rich repeat-containing G-protein-coupled receptors) and preventing the destabilization of the Wnt receptors. As a result, RSPOs act in concert with WNTs to promote the activation of the β-catenin-mediated intracellular signaling (46). Wolffian duct epithelium displays high WNT/β-catenin activity during epididymal coiling (47). When β-catenin is deleted specifically in the Wolffian duct epithelium before the region-specific patterning, the epididymis fails to coil at birth with significant reduction in proliferation and increases in cell death (47, 48). The defective morphogenesis (cystic formation) in the epididymal region is also observed when *Pkd1* (a receptor for mediating WNTs actions) is deleted in the epithelium (49, 50). The WNT receptor LGR4 is specifically expressed in the cranial region of fetal Wolffian ducts and postnatal epididymides (51, 52). Inactivation of *Lgr4* caused the cystic formation in the epididymis (51). Given the critical roles of WNT/β-catenin activities in the epithelium and AR actions in the mesenchyme in epididymal morphogenesis, mesenchymal AR might induce WNT ligands or activator as paracrine signals to regulate epididymal morphogenesis.

In the caudal region, another secreted ligand of TGFβ superfamily GDF7 is required for seminal vesicle growth, morphogenesis, and epithelial differentiation (**Figure 1**) (53). *Gdf7* is expressed in the seminal vesicle mesenchyme but its expression in other regions of Wolffian duct is not reported (53). *Gdf7^-/-^* males develop normal testis, epididymis, vas deferens and prostate; however, seminal vesicles are dramatically smaller and lack epithelial folding with decreased number of basal cells (53). In addition to *Gdf7*, AR actions in caudal Wolffian duct development are mediated by FGF10*/*FGFR2 signaling (54, 55). *Fgf10* (fibroblast growth factor 10) is a member in a gene family of generally extracellular signaling peptides, which are key regulators in organ development. The mesenchyme of the caudal Wolffian ducts expressed high *Fgf10*, which is increased by androgen treatment (**Figure 1**) (56). Global knockout of *Fgf10* led to the absence of seminal vesicle and distal vas deferens despite of normal testicular development (55). FGF10 has a high specificity for FGFR2, which is the major FGF receptor in the Wolffian duct epithelium (54). Wolffian duct-specific deletion of *Fgfr2* led to the degeneration of the caudal Wolffian ducts (54). FGF7 was also considered as a key mesenchymal paracrine growth factor for seminal vesicle development in *ex vivo* culture condition (57, 58). However, *Fgf7* knockout males does not display any reproductive phenotype (MGI: 95521), suggesting that *Fgf7* is dispensable in AR actions in promoting Wolffian duct development *in vivo*. These observations demonstrate that mesenchymal AR actions regulate region-specific morphogenesis *via* paracrine growth factors.

### How Are Mesenchymal AR Actions Affected by Epithelium-Derived Signals?

The crosstalk between the mesenchyme and epithelium during organogenesis is never a one-way street. The Wolffian duct epithelium-derived signal also has reciprocal inductive effects on the mesenchyme and can potentially influence mesenchymal AR actions. Wolffian duct epithelium specifically synthesizes a paracrine factor WNT9B, a member of the WNT ligands (45). *Wnt9b^-/-^* male embryos fail to form Wolffian duct derived organs at birth despite of normal testis development (59). The loss of the Wolffian duct-derived organ is also observed in male embryos that lack the direct upstream regulator of *Wnt9b, HNF1B* (60). In the *Hnf1β^-/-^* mice, the Wolffian duct are still partially present on E14.5 during sexual differentiation of Wolffian ducts. These observations suggest that Wolffian ducts in *Hnf1β^-/-^ or Wnt9b^-/-^* male embryos could be in process of degeneration on E14.5 when androgen actions are supposed to promote Wolffian duct stabilization. These observations indicate that signals derived from Wolffian duct epithelium are required for mesenchymal actions in stabilizing Wolffian ducts.

## Heterogeneity of Androgen Signaling in the External Genitalia

Androgen signaling is absolutely essential for sex differentiation of the external genitalia, making it susceptible to disruptions of androgen signaling as a result of genetic mutations or exposure to anti-androgenic chemicals. Defects of external genitalia are some of the most common birth defects in the world (61). Hypospadias, which is where the urethra exits not at the tip but along the shaft of the penis, is one of the most common birth defects of penis (61). Even though the requirement of androgen signaling in penis development has been known since the 1940’s, the causes for more than 70% of hypospadias cases remain unknown (62). One possible explanation for the unknown nature of hypospadias, is that penis formation is a product of hormone action and local cell-cell interactions. The intertwined actions of these pathways make the penis susceptible to genetic mutations and environmental exposure (62, 63). Researchers are only beginning to understand how all the cell types of the penis coordinate under the control of androgen to form a normal penis.

### Establishment of the Unique Androgen Responsive Programs in Penis Cell Populations

**Figure 2:**
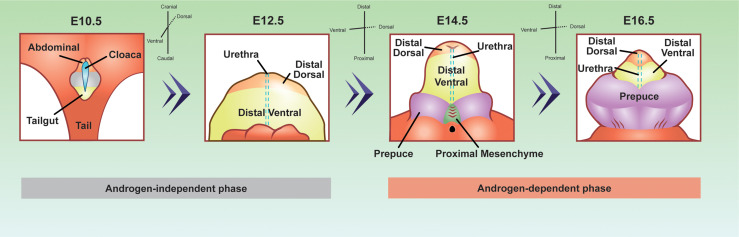
*Developmental origins of the penis cell populations in the embryonic mouse penis.* The colors represent conserved cell populations through time. Blue=cloacal/urethra epithelium, yellow=tailgut/distal ventral glanular mesenchyme, light orange=abdominal/distal dorsal glanular mesenchyme, purple=prepuce, green=proximal glanular mesenchyme, and grey= undefined penile cell population. The E10.5 depiction is looking at the lower half of the embryo, while the other time points are focused on the genitalia. E10.5 depiction was adapted and redrawn from (64). E12.5, 14.5, and 16.5 were adapted from whole mount, scanning electron microscope images from (65).

Penis development can be divided into two major phases, androgen-independent and androgen-dependent development (**Figure 2**) (66). The androgen-independent phase involves the transformation of the primitive cloaca into the genital tubercle, the precursor of the penis. At the end of embryonic development, the genital tubercle morphs into the penis that consists of the distal dorsal glans, distal ventral glans, proximal glans, prepuce, corporal bodies, and urethra epithelium (67). The distal dorsal glans, distal ventral glans, and urethral epithelium have distinct cellular origins. It is not elucidated whether the proximal glans, prepuce, and corporal bodies come from diverse cell origins, or a derived population of mesenchymal cells. It is the diverse origins of the cells and their cell states from the androgen-independent phase that imparts diverse androgen responsiveness during the androgen-dependent phase.

During the androgen-independent phase of fetal development, the cloaca arises between the hindlimbs of the embryo of both sexes (**Figure 2**). The cloaca is an ancestral structure present in most vertebrates where both the gastrointestinal and genitourinary tracts merge. Around mid-gestation or E10.5 in the mouse, the cloaca is a single chamber lined with epithelium, which is surrounded by mesenchymal cells. The mesenchymal cells contain two major populations that are associated with penis development, abdominal mesenchyme, and tailgut mesenchyme (**Figure 2**). The abdominal mesenchyme eventually differentiates into the distal dorsal glanular mesenchyme and the tailgut mesenchyme differentiates into the ventral glanular mesenchyme. The abdominal mesenchyme is specifically marked with the expression of *Alx4* gene while the tailgut mesenchyme is enriched for *Six1* and *Six2* genes (68, 69). Mutations of *Alx4* in mice lead to severe defects of the distal dorsal glans (68). Cultured genitalia with the abdominal mesenchyme removed, develop with an absent dorsal glans, displaying the importance of these progenitor cells in normal penis development (68). Mutations in the *Six1* and *Six2* genes cause defects in the ventral glans and urethra closure defects (69). *Six2* expressing cells at E13.5 and E14.5 comprise the distal ventral glans by E17.5 (64). These observations of *Alx4* and *Six2* expressing cells reveal that before the penis is formed, the distal dorsal glanular and distal ventral glanular mesenchyme have already established their unique identities in the cloaca.

Once the cloaca is separated into the gastrointestinal and genitourinary tracts around E13.5, the urethra epithelium appears along the ventral aspect of the genital tubercle. At this same time in development, two preputial buds form on either side of the established genital tubercle and the proximal glanular mesenchyme becomes present at the base of the genital tubercle (65). It is not clear if the preputial buds and the proximal glanular mesenchyme are derived from the abdominal or tailgut mesenchyme or if they originate from other sources.

The above described androgen-independent phase ends around E13.5, when androgen production by the testis starts. Simultaneous with androgen production, AR protein is most present in the cytoplasm of the urethral epithelium and the surrounding mesenchyme (70). One day later on E14.5, AR protein become localized in the nucleus of the proximal glanular mesenchyme, distal dorsal glanular mesenchyme, distal ventral glanular mesenchyme, prepuce, and urethral epithelium (71). From E14.5 through the rest of embryonic development, AR protein steadily increase in all mesenchymal cells of the penis and remains present in portions of the urethra epithelium (48). Disruptions in androgen signaling by fetal exposure to anti-androgenic chemicals or genetic knockouts of *Ar* cause severe cases of hypospadias, underlining the importance of androgen signaling in penis development.

### Androgen-Dependent Closure of the Proximal Urethra

**Figure 3:**
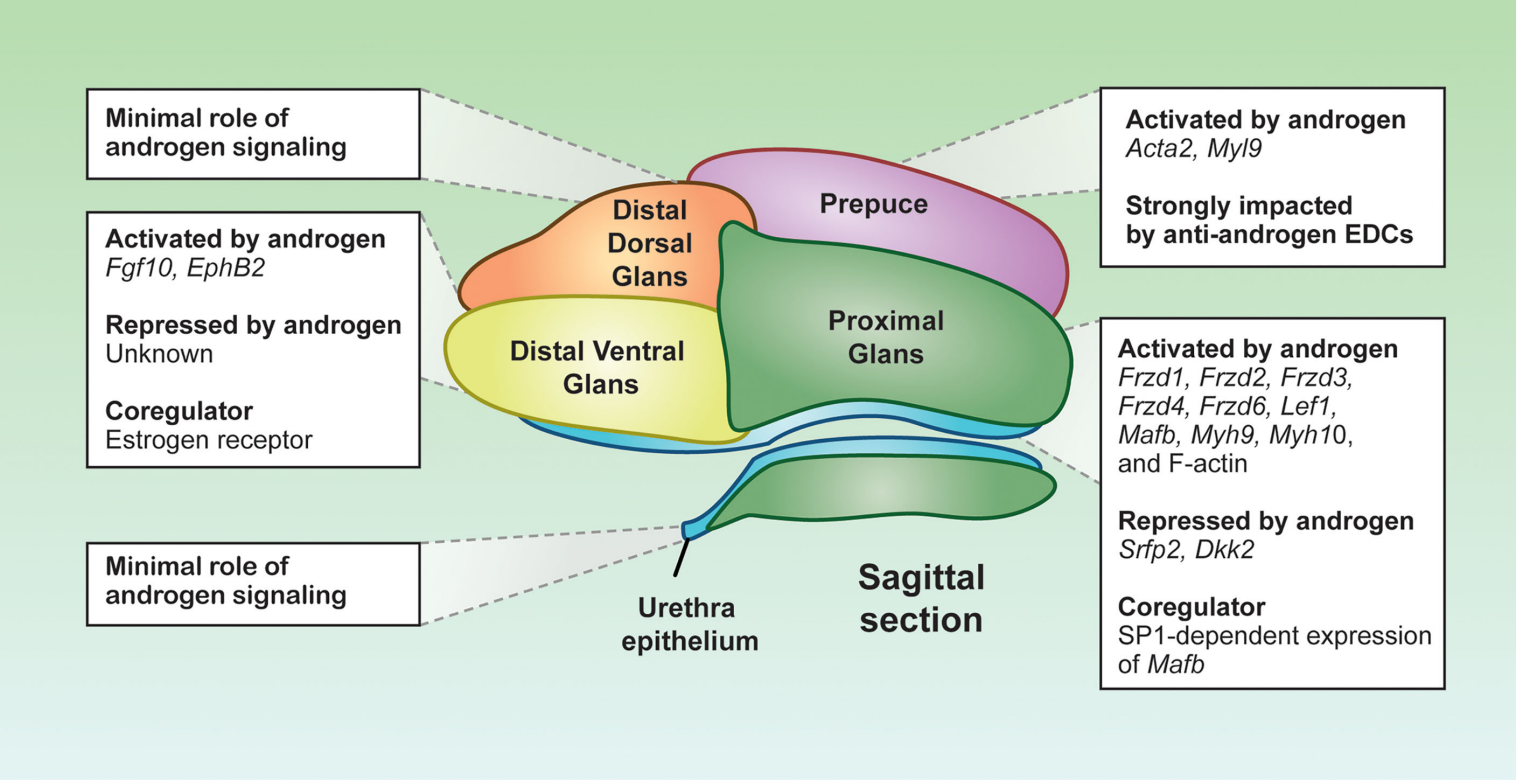
*Distinct androgen responsiveness of the penile cell populations.* Representative sagittal section depiction of an E16.5 mouse penis. Each color represents a distinct cell population, displaying the localization of the cell populations in the penis. Boxes are the androgen-related programs for each subpopulation. Depiction was generated from sagittal sections of mouse penises generated in the Yao laboratory.

The closure of the proximal urethra starts at E13.5 and E14.5 in the mouse embryo when the urethra is still an open sulcus at the base of the penis (72). By E15.5, the proximal portion of the urethra starts to close and form an internalized tube (72). To accomplish this event, extensive androgen-dependent communication occurs between the urethral epithelium and proximal glanular mesenchyme. Although the urethral epithelium expresses *Ar* mRNA and protein throughout development (67, 73), AR in the urethral epithelium is not required for urethral closure and proper penis formation (70, 74) (**Figure 3**). Conversely, inactivation of *Ar* in the surrounding mesenchyme causes severe cases of hypospadias, indicating the necessity of mesenchymal androgen signaling for urethra closure (70, 74–76).

The proximal glanular mesenchyme is a group of mesenchymal cells that surrounds the urethra. These mesenchymal cells are sites of strong androgen signaling, largely due to abundant expression of *Srd5a2* that convert testosterone to the more potent dihydrotestosterone (77). As a result, dihydrotestosterone is twice as concentrated in the proximal glanular mesenchyme compared to other areas of the penis (77). The abundance of androgens facilitates the expression of key androgen responsive genes in the proximal glanular mesenchyme, like the transcription factor *Mafb* and the AR co-chaperone *Fkbp5*, both essential for urethra closure (75, 78). The expression of *Mafb* is not only dependent on AR binding to the ARE, but also requires the co-regulator SP1 to bind at the *Mafb* enhancer element. Loss of SP1 binding motif in the *Mafb* enhancer element results in severe urethra closure defects (79) (**Figure 3**). The combination of the high concentration of androgens and unique presence of co-activator provide the proximal glanular mesenchyme with the unique ability to orchestrate urethra closure.

Two possible mechanisms of androgen-dependent urethra closure are proposed between the proximal glanular mesenchyme and the urethral epithelium. One is through mechanical forces that help push the urethra closed, and the other is cell-cell interactions through morphogens that instruct structural changes of the epithelium and mesenchyme. Androgen signaling in the proximal glanular mesenchyme induces contractility and expression of several muscle related genes such as myosin heavy chain 10 (*Myh10*), *Myh9*, and F-actins (80, 81). Myosin and F-actin provide the cells the capacity to contract and impart physical lateral forces on surrounding cell populations. Inactivation of *Myh9/10* in the proximal glanular mesenchyme in mice causes severe urethra closure defects arise due to the loss of capability to exert physical forces on the urethra (80) (**Figure 3**).

The contractility capability of the proximal glanular mesenchyme is not only induced directly by androgens, but also by androgen-dependent morphogen pathways from the urethra and proximal mesenchyme. The WNT signaling pathway, identified through the Gene Ontology analyses on the proximal mesenchyme, is specifically enriched in the proximal glanular mesenchyme of the penis (67). The main WNT ligands, *Wnt5a, Wnt6, Wnt7a, Wnt10a* and *Wnt2*, are expressed throughout the penis, with *Wnt5a* being the main effector in the proximal glanular mesenchyme (82, 83). Removal of *Wnt5a* expression in the proximal glanular mesenchyme disrupts the contractility phenotypes of the proximal glanular mesenchymal cells and caused severe hypospadias-like phenotypes in the mice (84). However, *Wnt5a* expression does not seem to be controlled directly by androgens (84). Several of the WNT receptors and transcription factors, in contrast to *Wnt5a*, are induced by androgen signaling (85). The WNT receptor, FRZD6, and the WNT-related transcription factor such as LEF1 are significantly enriched in the male proximal mesenchyme, when compared to the female (85). Exposure of female embryos to androgen (methyltestosterone) increases expression of both FRZD6 and LEF1 proteins (86). Other WNT receptor gene expression, *Frzd1*, *Frzd2*, *Frzd3*, and *Frzd4*, are also significantly elevated in methyltestosterone exposed female (85). In addition, the WNT ligands and receptors, WNT inhibitors, *Dkk2* and *Sfrp2*, are higher in female genitalia compared to males, and the expression of these WNT inhibitors significantly lessen with androgen exposure (**Figure 3**) (74). These findings reveal that closure of the proximal urethra requires a complex WNT signaling crosstalk between the proximal glanular mesenchyme and urethral epithelium in an androgen-dependent fashion.

### A Role for Estrogen Receptor Coordination With AR in Distal Urethra Closure

The closure of the urethra occurs in a proximal (base) to distal (tip) fashion along the ventral aspect of the penis (87). At E16.5 in the mouse embryo, the urethra is largely closed in the proximal portion, but remains open in the distal portion of the penis (65). The distal ventral glanular mesenchyme subsequently interacts with the urethra to form a tube and complete the closure of the urethra. Similar to the proximal glans, the distal ventral glans express myosin light chain 12a (*Myl12a*) and *Myl6* and is enriched with actin-cytoskeleton signaling pathway components, suggesting that it may exert similar mechanical forces on the distal urethra (67). The expression of these genes are also elevated in the male distal ventral glans compared to the female, an indication that they could be controlled by androgens.

Other than the androgen signaling, the distal ventral glans has the highest expression of estrogen receptor (ER) compared to the rest of the penis (67). Estrogen receptor and AR can be both antagonistic and agnostic to one another depending on hormone levels and tissue types (**Figure 3**) (88). For example, the estrogenic chemicals such as estradiol benzoate, diethylstilbesterol, and 17β-estradiol, cause only distal hypospadias (70, 89, 90). The degree of defects in urethra closure drastically differs among different anti-androgen exposure, which induces severe hypospadias where the urethra exits at the base of the penis (91). ER may be antagonizing AR at hormone response elements along the DNA within the distal ventral glans. Or estrogen-dependent signaling could result in gene expression that inhibits androgen-dependent signals (**Figure 3**). Although aberrant exposure to exogenous estrogens causes distal hypospadias, the ER signaling pathway appears to be essential for normal penis development in some capacity. Estrogen receptor α or *Esr1* knockout mice develop mild hypospadias in adulthood, similar to estrogen exposed mice (90). These data suggest that a properly tuned estrogen signaling in the penis is essential, where too little or too much estrogen is detrimental to penis development.

This proposed role of estrogen signaling in penis development is likely required to modulate appropriate morphogen expression. One such morphogen is fibroblast growth factor or FGF. In the fetal mouse penis, FGF10 ligand and its receptor FGFR2IIIβ are uniquely expressed in the distal ventral glans (92, 93). Global knockout of *Fgf10* causes severe hypospadias with no urethra closure occurring throughout the penis (92, 93). *Fgf10* is tightly linked to both androgen and estrogen signaling in many hormone responsive tissues (**Figure 3**). In the ventral prostate and the uterus, estrogen exposure and *Esr1* are essential for establishing appropriate *Fgf10* expression (94, 95). In the penis, *Fgf10* is induced with androgen supplementation and not much is known about it estrogen responsivity (93). The interactions between androgen and estrogen receptor signaling on gene expression remains to be elucidated in the distal ventral glans.

### Androgen-Dependent Encapsulation of the Glans by the Prepuce

The prepuce in the male mouse embryo originates as two bilateral buds on the lateral sides of the glans at the onset of androgen-dependent development, followed by extensive outgrowth after androgen production in the fetal testis. Soon after the preputial buds form on the penis, the prepuce begins to express *Ar* (67). In the male embryo, the cells within the prepuce have a stretched, migratory phenotype with a clear directionality pointing toward the urethra (80). The prepuce starts to fuse along the midline of the penis at E15.5. Closely following the process of urethra closure, the prepuce eventually fuses to the tip of penis and fully encapsulates the glans and urethra.

Inactivation of the androgen signaling, either through global *Ar* knockout or exposure to environmental chemicals, result in severe aplasia of the prepuce and a lack of fusion along the ventral aspect of the penis (70, 91). The majority of hypospadias cases are accompanied with preputial defects (96, 97). When external genitalia are cultured without androgens, preputial cell migration does not occur (80). The migratory loss could be due to the diminished expression of smooth muscle actin (*Acta2*) and myosin light chain 9 *(Myl9*), which are both expressed in the developing prepuce (67) (**Figure 3**). The correlations between preputial fusion and urethra closure suggest a role of the prepuce in urethra closure.

### Estrogen and Androgen-Dependent Differentiation of the Corporal Bodies

The corporal bodies of the penis are mesenchymal condensations that arise within the glans of the penis later in development around E17.5-E18.5 in mice (98). The corporal bodies are column condensations that run throughout the entire penis. In adult life, the corporal tissues are essential for erectile function. Mice have three major corporal tissues, the corpus cavernosum, corpus cavernosum glandis, and corpus cavernosum urethrae (99). The corpus cavernosum is located in the internal portion of the penis and connects to the penis bone, which maintain extrusion of the penis from the prepuce during erection (98). Where the corpus cavernosum ends close to the proximal end (or base) of the penis, the external portion of the penis (glans) begins. Within the glans lies the corpus cavernosum glandis as a muscular ring around the outer edge of the penis. Toward the center of the glans is where the corpus urethrae located. The corpus urethrae helps maintain an open urethra during copulation (98). Development of the three corporal tissues are androgen-dependent in mice. In the *Tfm* mice, where AR is nonfunctional, the corporal tissues are drastically reduced (100). The corpus cavenosum glandis and corpus urethra express *Ar*, *Esr1*, and *Esr2*. *Esr1* knockout mice fail to develop the corpus cavernosum glandis, while the *Esr2* knockout mice fail to develop the corpus urethra (101). In humans, both corporal tissues express AR and ESR2 (102), suggesting a potential interplay between the androgen and estrogen signaling pathways.

### Dorsal Mesenchyme of the Penis as an Androgen Insensitive Cell Population

In contrast to the tissues in the proximal and ventral penis, the distal dorsal glans seems to have weak androgen responsiveness and plays a minor role in the androgen-dependent processes of penis development. Unique to the male mice, a portion of the distal dorsal glans differentiates postnatally into a cartilaginous mating protuberance, or the male urogenital mating protuberance (MUMP) (99), which is not found in female mice nor in the human (99). In addition to the MUMP, the distal dorsal glans is involved in the overall growth and size of the penis. Genetic mutations of dorsal glans gene *Inhba* in mice caused the abnormally enlarged penis (103). The role of androgen signaling within the distal dorsal glans remains to be determined.

## Commonality of AR- Mediated Male Sexual Differentiation of Wolffian Duct and External Genitalia

### Androgen Action Must Be Imposed Within a Short Developmental Period Known as Masculinization Programming Window

**Figure 4:**
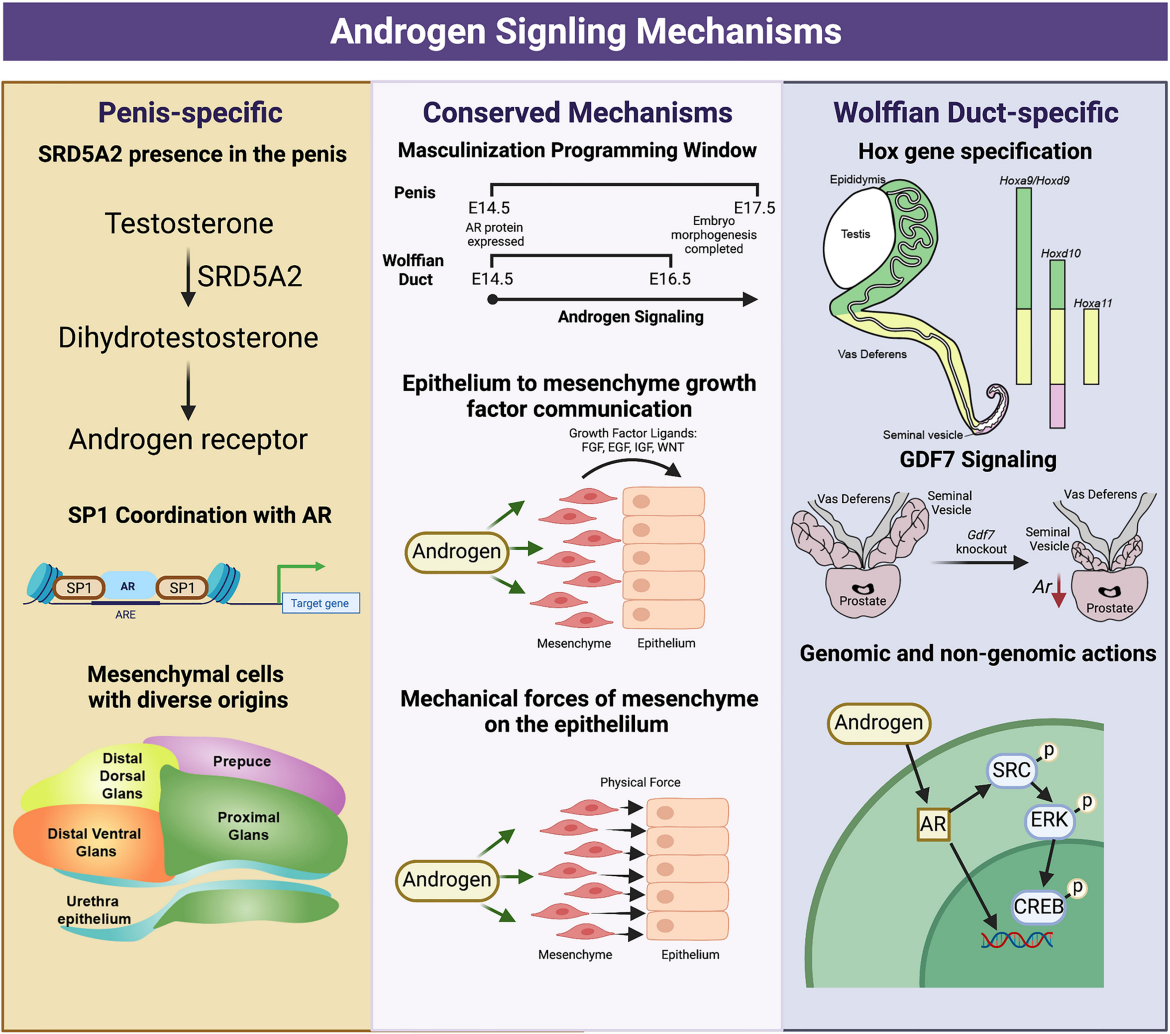
*Divergent and conserved androgen signaling mechanisms in the fetal development of the Wolffian Duct and penis.* The Wolffian Duct and penis have conserved androgen signaling mechanisms that include a window of masculinization, growth factor signaling (EGF,FGF, and WNT) and mechanical forces from the mesenchyme to the epithelium. However, these two organs also possess divergent androgen signaling mechanisms. In the penis, *Srd5a2* is present and converts testosterone to dihydrotestosterone. AR and SP1 interact along the DNA to elicit both gene expression and chromatin modifications, and the mesenchymal cells of the penis have a more diverse community of mesenchymal cells from different development origins. In the Wolffian duct, mesenchyme in different regions expresses region-specific *Hox* genes that govern the regionalization of the Wolffian ducts. The differentiation of the cranial Wolffian duct to the seminal vesicle depends on *Gdf7*. Wolffian duct development requires both genomic and non-genomic androgen signaling while non-genomic androgen signaling is dispensable in the penis development. The figure summarizes data and reviews from (16, 18, 27, 28, 53, 108) on Wolffian ducts and (67, 77, 79, 80, 91, 93, 104, 106) on external genitalia. Figure Created with BioRender.com

The action of AR must be imposed within a specific fetal programming window. For the Wolffian ducts, the masculinization programming window is E15.5–E17.5 in rat (104), presumably E14.5-16.5 in mouse given that mouse development is generally 1 day behind that of rat (86), and predicted to be approximately 8-12 weeks of gestation in humans (104). The critical window for the Wolffian duct overlaps considerably with the critical window for the penis, with timeframes ranging from E15.5-18.5 in the rat (104), E14.5-E17.5 in the mouse (105), and 8-12 weeks of gestation in the human (104) (**Figure 4**). Only within this programming window, disruption of androgen actions by AR antagonist flutamide results in partial or complete absence of Wolffian ducts by adulthood in rat. AR action-associated endpoints subside in the presence of flutamide, including cell proliferation, epididymal coiling, epithelial vimentin expression, and smooth muscle actin expression in the Wolffian duct inner stroma (106). Similarly, only within this window does impaired androgen action result in cryptorchidism and hypospadias (104). The most severe cases and highest incidence of hypospadias occurs with flutamide exposure between E15.5 and E16.5 (105). Flutamide exposures before E15.5 can result in severe hypospadias, but low incidence rates, while exposures after E16.5 result in both mild hypospadias cases and low incidence rates (70, 91). The same degree of hypospadias severity was shown to be true for AR knockouts at E14.5 and E17.5. The masculinization window also exists in the female embryos. For example, seminal vesicle formation in female rats is induced by androgen exposure during E15.5-17.5, the masculinization program window but not by exposure during E19.5-E21.5 (104). Female rats develop complete Wolffian ducts and penis only when they are exposed to androgens during this window. The window is tightly regulated, where exposure to androgens before or during the masculinization window does not advance or extend the timing of the critical window (107). The molecular determinants of opening/closing the programming window are not completely understood. It appears that the induction of AR protein in Wolffian duct and penile tissues (20) coincides with the opening of the window.

### Androgen Action in the Mesenchyme Regulates Epithelial Morphogenesis

Extensive studies have demonstrated the essential role of AR action in the mesenchyme of Wolffian ducts and penis (**Figure 4**). Epithelial ablation of AR in the Wolffian duct or the penis do not affect maintenance and coiling of the Wolffian duct (20), nor urethra closure in the penis (74). The functional significance of the mesenchymal AR in penis development has been demonstrated in mesenchymal specific AR knockouts mice where severe urethral and penile defects were observed (74). Although the consequences of delating the mesenchymal AR has not yet been determined in Wolffian duct development *in vivo*, classic tissue recombinants studies provide evidence that the mesenchymal AR actions governs androgen-induced epithelial morphogenesis (25) (See the section *Mesenchymal AR actions are critical for Wolffian ducts differentiation*).

### Androgen Actions Induce Epithelial Morphogenesis Through Both Growth Factors and Mechanical Forces

In response to androgens, the mesenchyme produces multiple mesenchymal growth factors (FGFs, EGF, and WNT) that mediate mesenchymal AR actions in controlling survival and differentiation of Wolffian ducts and penis (18, 74, 109). Aside from growth factor signaling, mesenchymal AR can also regulate epithelial morphogenesis by inducing expression myosin and actin related genes, which induce mechanical forces on the epithelium (108). During Wolffian duct morphogenesis, inner mesenchymal cells differentiate into smooth muscle on E16.5, the initiation of Wolffian duct coiling. The smooth muscle is known to produce mechanical resistance. Blocking α-smooth muscle actin (aSMA), a marker of smooth muscle differentiation, significantly reduces tubule folding without affecting cell proliferation in the tubule or the length of the epididymal tubule (108). In the penis, androgen induced expression of myosin-related genes, *Myh10* and actomyosin contractility in the proximal mesenchyme. *In vivo* genetic ablation of both Myh10 and Myh9 in the bilateral or pharmacological inhibition of actomyosin contractility in *ex vivo* slice culture system induced defective urethral masculinization (80). These results suggest that mesenchyme-derived mechanical force is the other mechanism by which androgen induce epithelial morphogenesis.

## Summary

Although AR is a singular transcription factor, it can fill many roles throughout Wolffian duct and male external genitalia development. In both organs, plenty of research has identified androgen responsive programs, but there many questions about the molecular drivers of AR pleiotropy. Future studies that conduct cell type-specific investigations of AR co-regulator interactions, chromatin accessibility of AREs, and multi-organ androgen signaling conservation will bring us drastically closer to understanding the prevalence of androgen-related human birth defects.

## Author Contributions

CA, FZ, and H-CY were responsible for creating figures, writing content, and editing the manuscript. All authors contributed to the article and approved the submitted version.

## Funding

Intramural Research Program of National Institute of Environmental Health Sciences Z01-ES102965 (H-CY). National Institute of Child Health and Development R00-HD096051 (FZ).

## Conflict of Interest

The authors declare that the research was conducted in the absence of any commercial or financial relationships that could be construed as a potential conflict of interest.

## Publisher’s Note

All claims expressed in this article are solely those of the authors and do not necessarily represent those of their affiliated organizations, or those of the publisher, the editors and the reviewers. Any product that may be evaluated in this article, or claim that may be made by its manufacturer, is not guaranteed or endorsed by the publisher.
